# Antibacterial Potential of *Jatropha* sp. Latex against Multidrug-Resistant Bacteria

**DOI:** 10.1155/2020/8509650

**Published:** 2020-08-27

**Authors:** Muhammad Evy Prastiyanto, Prayoda Deri Tama, Ninda Ananda, Wildiani Wilson, Ana Hidayati Mukaromah

**Affiliations:** ^1^Microbiology Laboratory, Department of Medical Laboratory Technology, Universitas Muhammadiyah Semarang, Kedungmundu Raya Street 18, Semarang 50273, Indonesia; ^2^Indonesian Medicinal Laboratory Science, Kedungmundu Raya Street 18, Semarang 50273, Indonesia; ^3^Bachelor Program Department of Medical Laboratory Technology, Faculty of Nursing and Health Science, Universitas Muhammadiyah Semarang, Kedungmundu Raya Street 18, Semarang, Indonesia; ^4^Chemistry Laboratory, Department of Medical Laboratory Technology, Universitas Muhammadiyah Semarang, Kedungmundu Raya Street 18, Semarang 50273, Indonesia

## Abstract

**Objective:**

This study was aimed to evaluate the antibacterial activity of the latex of three species members of *Jatropha* (*J. curcas*, *J. gossypilofia* Linn., and *J. multifida*) against *methicillin-resistant Staphylococcus aureus* (MRSA), extended-spectrum beta-lactamase- (ESBL-) producing *Escherichia coli* and ESBL-producing *Klebsiella pneumonia,* carbapenemase-resistant *Enterobacteriaceae* (CRE)-*E. coli, K. pneumoniae*-carbapenemase (KPC), and carbapenemase-resistant *Pseudomonas aeruginosa* (CRPA).

**Method:**

The antibacterial activities were calculated based on the inhibition zones using the Mueller–Hinton agar diffusion method, minimum inhibitory concentration (MIC) using Mueller–Hinton broth in a microdilution method, and minimum bactericidal concentration (MBC) using blood agar plate.

**Results:**

The latex of *Jatropha* showed antibacterial activities against the MRSA and CRPA. All latex of *Jatropha* appeared to have the antibacterial activities against MRSA and CRPA in the diffusion method (20.4–23.7 mm and 12–15 mm), MIC (0.19–6.25%, and 25%), and MBC (0.39–12.5% and 50%). Phytochemical screening of latex indicated the presence of flavonoids.

**Conclusions:**

The latex of *J. curcas*, *J.* g*ossypilofia* Linn., and *J. multifida* has the potential to be developed as antibacterial agents, especially against MRSA and CRPA strain, but further in vivo research and discovery of the mode of its action are required to shed the light on the effects.

## 1. Introduction

Bacterial resistance to antibiotics has become a serious problem in the world, including in Indonesia. This is a factor in the high mortality rate and continues to increase every year [1]. Inappropriate use of antibiotics is one of the causes of antibiotic resistance [2]. The emergence of multidrug-resistant (MDR) bacteria causes treatment ineffectiveness [3]. This has a devastating effect on patients suffering from MDR bacterial infections, increasing the treatment costs, which become increasingly high and difficult to afford and causing prolonged illness and even death [4]. One alternative to overcome these problems is to use natural ingredients such as materials from fungi [5] and plants [6], one of which is *Jatropha*.

Our study aimed to investigate the antibacterial activities of *Jatropha* latex against MDR bacteria. In this study, we used three species of *Jatropha* plants to measure the antibacterial activities against MDR bacteria such as *methicillin-resistant Staphylococcus aureus* (MRSA), extended-spectrum beta-lactamase (ESBL)-producing *Escherichia coli*, ESBL-producing *Klebsiella pneumonia*, carbapenemase-resistant *Enterobacteriaceae* (CRE)-*E. coli, K. pneumoniae*-carbapenemase (KPC), and carbapenemase-resistant *Pseudomonas aeruginosa* (CRPA). Three types of latex used in this study were *Jatropha curcas*, *Jatropha gossypilofia* Linn., and *Jatropha multifida*.

## 2. Methods

### 2.1. Collection of Latex

*Jatropha* was collected from the field of Universitas Muhammadiyah Semarang in Semarang at the rainy season in January 2019. The collected plants were identified and classified according to the types of Herbarium Semarangense at the Department of Biology, Universitas Negeri Semarang. The latex of *J. curcas*, *J.* g*ossypilofia* Linn., and *J. multifida* was collected by cutting the stems. The latex was stored in sterile dark bottles.

### 2.2. Materials

The used materials were Autoclave (*Hirayama* HICLAV HV-25), Incubator (WTC Binder), biological safety cabinet (*Labconco* Purifier Class II Biosafety Cabinet), freezer (Electrolux), densimat (*Biomerieux*), Mueller Hinton Agar (MHA), Mueller Hinton Broth (MHB), Blood Agar Plate (BAP), *McFarland*, MRSA, ESBL-producing *Escherichia coli*, ESBL-producing *Klebsiella pneumonia,* CRE-*E. coli*, KPC, and CRPA that were obtained from the Medical Microbiology Laboratory of dr. Kariadi Central Hospital, Semarang, Central Java, Indonesia. MDR isolates were identified, and the susceptibility patterns were obtained using Vitek^®^MS (*BioM´erieux*).

### 2.3. Bacterial Preparation

The tested organisms were subcultured BAP for 24 h at (35 ± 2)°C. The colonies were inoculated in a normal saline solution. The bacteria cell suspensions were homogenized and adjusted to 0.5 McFarland standards (1.5 × 10^8^ CFU/mL) using a densimat.

### 2.4. Antibacterial Assay of Latex

#### 2.4.1. Agar Well Diffusion Assay

The antibacterial activities of latex were evaluated using agar well diffusion assay [7, 8]. In this method, 100 *μ*L of each test organism, which was equivalent to a 0.5 McFarland standard, was inoculated on the MHA. Then, it was spread on to the surface of the agar using a sterilized glass spreader. After 10 minutes of inoculation, the wells were prepared using a sterilized steel cork borer (1 cm in diameter). Wells were made on each plate, out of which three wells were loaded with each latex. Each test was done in triplicate. All the plates were then incubated aerobically at 35 ± 2°C for 16–20 h. The antibacterial activities were evaluated by measuring the diameters of zones of inhibition (mm) against the test organism.

#### 2.4.2. Minimum Inhibitory Concentration (MIC) and Minimum Bactericidal Concentration (MBC)

The MIC of *Jatropha* latex was determined using Mueller–Hinton broth microdilution [9]. MIC determination was performed by a serial dilution technique using 12-well microtiter plates. MHB (100 *μ*L) was placed into the well/plate and *Jatropha* latex (100 *μ*L) in the dilution series. 10 *μ*L bacterial cell suspensions were placed in each well/plate. Microplates were incubated aerobically at 35 ± 2°C for 16–20 h. The lowest concentrations without visible growth completely inhibited the bacteria (MIC) [10]. The MBC was defined as the lowest concentration of the extract that did not allow any growth [10]. The MBC of *Jatropha* latex was determined following the methods described by [11] with slight modifications. Wells were subcultured using a 10 *μ*L inoculating loop on to a 5% sheep BAP at (35 ± 2)°C for 16–20 h incubation.

### 2.5. Phytochemical Screening

The *Jatropha* latex was screened for the presence of different classes of secondary metabolites, including alkaloids and flavonoids, using previously described methods [12].

## 3. Results

### 3.1. Agar Well Diffusion Assay

**Figure 1:**
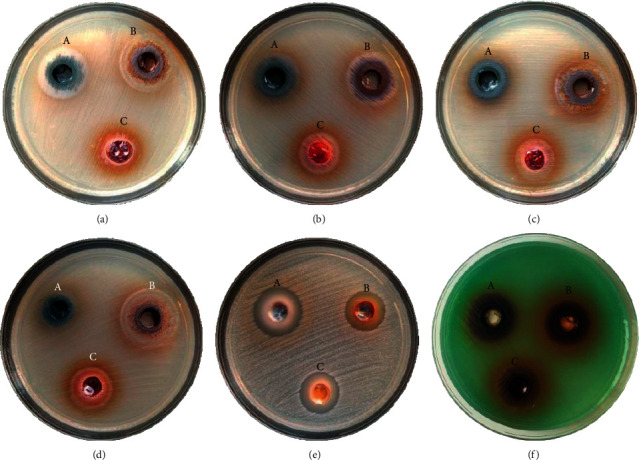
Zones of inhibition of *Jatropha* latex against MDR bacteria: (a) ESBL-*Klebseilla pneumonia*, (b) ESBL-*E. coli*, (c) CRE *E. coli*, (d) KPC, (e) MRSA, (f) CRPA, (A) *J. curcas*, (B) *J. gossypilofia* Linn., and (C) *J. multifida*.

The antibacterial activities of three *Jatropha* species were examined in vitro using the diffusion and dilution of six MDR bacteria.  Figure 1 shows that all latex exhibited inhibition zones against MRSA and CRPA. *J. curcas* showed a greater inhibitory zone diameter of 23.7 mm against MRSA and 15 mm against CRPA (Table 1).

### 3.2. Minimum Inhibitory Concentration (MIC) and Minimum Bactericidal Concentration (MBC)

**Figure 2:**
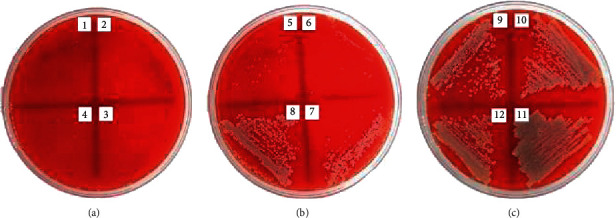
MBC value of *J. curcas* against MRSA at 6.25%.

**Figure 3:**
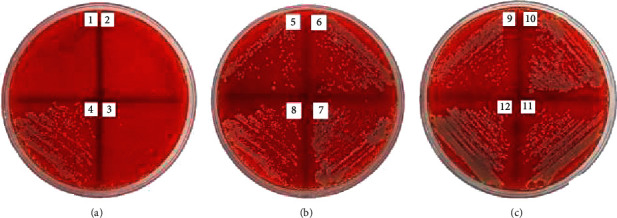
MBC value of *J. gossypilofia* Linn. against MRSA at 12.5%.

**Figure 4:**
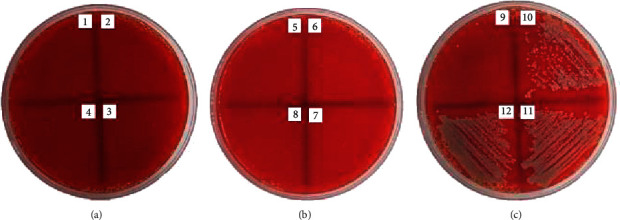
MBC value of *J. multifida* against MRSA at 0.39%.

**Figure 5:**
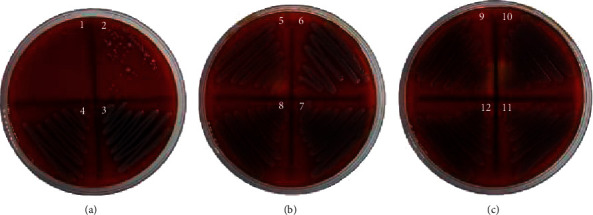
MBC value of *J. curcas* against CRPA at 50%.

**Figure 6:**
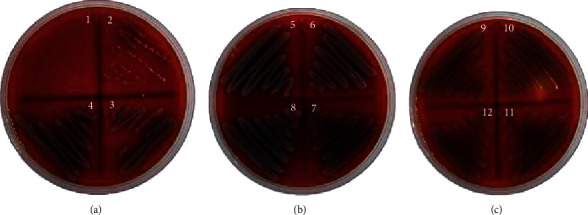
MBC value of *J. gossypilofia* Linn. against CRPA at 50%.

**Figure 7:**
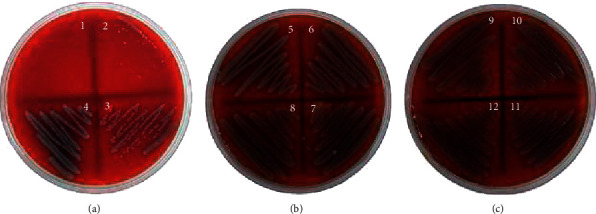
MBC value of *J. multifida* against CRPA at 50%.

The antibacterial activities of the *Jatropha* latex (*J. curcas*, *J. gossypilofia* Linn., and *J. multifida*) were assayed in vitro by the agar microdilution method against MRSA and CRPA.  Table 2 demonstrates that among the three *Jatropha* latex, *J. multifida* exhibited the smallest value of MIC and MBC against MRSA (0.19% and 0.39%). Figures 2–4 show that *J. curcas* and *J. gossypilofia Linn*. had the same activities against MRSA.  Table 2 displays that all latex exhibited the same value of MIC and MBC against CRPA (25% and 50%). Figures 5–7 exhibit that *J. curcas*, *J. gossypilofia* Linn., and *J. multifida* had the potential to be developed as antibacterial agents for MRSA and CRPA strains.

### 3.3. Phytochemical Analysis

The secondary metabolites are presented in Table 3. Flavonoids are present in all *Jatropha* latex.

## 4. Discussion

The research for new antibacterial agents from a natural or organic source has become a very important endeavor, considering the escalating levels of antibiotic resistance. One of the efforts in this research is focused on the use of *Jatropha* latex, which is widely available. Traditional medicine has been practiced worldwide, including in Indonesia, for centuries.

The agar well diffusion and microdilution methods were used in the present study due to their routine use as a quantitative method in clinical laboratories. In the agar well diffusion method, 100 *μ*L of each MDR organism, equal to a 0.5 McFarland standard, was inoculated on the MHA. The wells were prepared using a sterilized teel cork borer (1 cm diameter). Wells were made on each plate, out of which three wells were loaded with each of latex. All the plates were then incubated at 35 ± 2°C for 16–20 h. Antibacterial activities were evaluated by measuring the diameters of zones of inhibition (mm) against the MDR organism. In microdilution method, susceptibility test in 12 well microtiter plates contained various concentrations (50, 25, 12.5, 6.25, 3.12, 1.56, 0.78, 0.39, 0.19, 0.09, 0.04, and 0.02%) of *Jatropha* latex. Then, the standardized numbers of MDR bacteria were inoculated into the wells of the microtiter plates and incubated at 35 ± 2°C for 16–20 h. The MIC value was observed as the lowest concentration where no viability was detected in the wells after incubation. Wells in MIC assays were subcultured using a 10 *μ*L inoculating loop onto a BAP at 35 ± 2°C for 16–20 h incubation. The MBC was defined as the lowest concentration of the extract that did not permit any growth.

The latex of *Jatropha* showed antibacterial activities against the MRSA and CRPA. All latex of *Jatropha* showed the antibacterial activities against MRSA and CRPA in the diffusion method (20.4–23.7 mm and 12–15 mm), MIC (0.19–6.25% and 25%), MBC (0.39–12.5% and 50%). These results are following the research report by Hernandez-Hernandez [13] that the latex of *Jatropha neopauciflora* Pax demonstrated inhibition against *S. aureus*. In another study, the latex of *Jatropha curcas* displayed potent antimicrobial activity against *Pseudomonas aeruginosa* [14]. Phytochemical screening of latex showed the presence of flavonoids. These flavonoid compounds have been reported to be used by plants for protection against bacteria and are responsible for antimicrobial activity [15]. The latex of *J. curcas*, *J. gossypilofia* Linn., and *J. multifida* has the potential to be developed as anti-MDR-bacterial agents, especially against MRSA and CRPA strain, but further in vivo research is still needed to explicate on the effects.

## 5. Conclusion

The latex of *J. curcas*, *J.* g*ossypilofia* Linn., and *J. multifida* is prospective for development as an antibacterial agent, especially against MRSA and CRPA strain, but advanced in vivo research and discovery of the mode of its action are necessary to throw light upon the effects.

## Figures and Tables

**Table 1:** Diameters of zones of inhibition of Jatropha latex against MDR bacteria (mm).

Sample	ESBL-producing *K. pneumoniae*	ESBL-producing *E. coli*	CRE-*E. coli*	KPC	MRSA	CRPA
*J. curcas*	0	0	0	0	23.7	15
*J. gossypilofia* Linn.	0	0	0	0	20.6	13
*J. multifida*	0	0	0	0	20.4	12

**Table 2:** MIC of Jatropha latex against MDR bacteria (%).

Sample	MIC		MBC	
	MRSA	CRPA	MRSA	CRPA
*J. curcas*	3.12	25	6.25	50
*J. gossypilofia* Linn.	6.25	25	12.5	50
*J. multifida*	0.19	25	0.39	50

**Table 3:** Results of phytochemical analysis of Jatropha latex.

Sample	Secondary metabolites		
	Flavonoid	Terpenoids	Alkaloids
*J. curcas*	+	−	−
*J. gossypilofia* Linn.	+	−	−
*J. multifida*	+	−	−

## Data Availability

The data used to support the findings of this study are included within the article.
